# *Astragalus* Polysaccharides Induce Immunogenic Cell Death in Melanoma: A Mechanism Mediated by cGAS/STING Activation via Intratumoral Microbiota Modulation

**DOI:** 10.1002/cam4.71797

**Published:** 2026-04-14

**Authors:** Xi Qiao, Guiqing Ding, Qijin Lu, Yuxin Chen, Xiaohan Wang, Yuanhua Wang, Jinyun Ma, Xiaodong Cheng

**Affiliations:** ^1^ Institute of Clinical Immunology, Yue‐Yang Hospital of Integrative Medicine Shanghai University of Traditional Chinese Medicine Shanghai China

**Keywords:** *Astragalus* polysaccharides, cGAS‐STING, immunogenic cell death, intratumoral microbiota, melanoma

## Abstract

*Astragalus* polysaccharides (APS) exhibits potent immunomodulatory properties, yet its interplay with intratumoral microbiota in driving antitumor immunity remains elusive. Herein, we demonstrate that APS suppresses melanoma growth and prolongs survival by inducing immunogenic cell death (ICD) via the cGAS‐STING pathway. Inhibition of cGAS‐STING abrogated APS efficacy. Notably, depletion of intratumoral microbiota using antibiotics blunted APS‐mediated cGAS‐STING activation and immune responses, confirming a microbiota‐dependent mechanism. 5R 16S rRNA sequencing identified *Pseudomonas* enrichment as a distinct signature of APS treatment. Crucially, administration of *Pseudomonas* sterile supernatant was sufficient to activate cGAS‐STING signaling, induce ICD, and inhibit tumor growth in microbiota‐depleted mice. We propose that APS acts as an upstream modulator that enriches *Pseudomonas,* creating a microenvironment where bacterial secretions trigger the cGAS‐STING pathway to induce ICD. These findings provide a novel theoretical basis for microbiota‐targeted immunotherapies.

## Graphical abstract

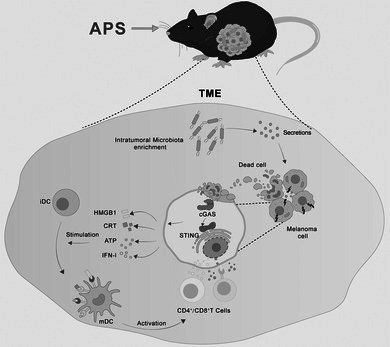

**Abbreviations**
ABX: antibiotic cocktail
ANXA1: Annexin A1
APC: antigen‐presenting cells
APS: *Astragalus* polysaccharides
ATP: adenosine triphosphate
CRT: calreticulin
DAMPs: Dmage‐associated molecular patterns
DCs: dendritic cells
ELISA: enzyme‐linked immunosorbent assay
FBS: fetal bovine serum
GC–MS: gas chromatography–mass spectrometry
HMGB1: high‐mobility group box 1
ICD: immunogenic cell death
IFN‐I: type I interferon
LPS: lipopolysaccharide
LTA: lipoteichoic acid
mtDNA: mitochondrial DNA
MTT: methylthiazolyldiphenyl‐tetrazolium bromide
PCA: principal component analysis
PD‐1: programmed death 1
RLU: relative luminescence units
SMRF: short multiple regions framework
SPF: specific pathogen‐free
TDLNs: tumor‐draining lymph nodes

## 1 Introduction

Melanoma, the most aggressive skin cancer, accounts for over 60% of mortality in skin cancer cases, despite comprising only 5% of cases [1]. The high propensity of melanoma to metastasize, coupled with the limited efficacy of current treatments like dacarbazine and programmed death 1 (PD‐1) inhibitors [2], underscores the urgent need for novel therapeutic strategies. The low immunogenicity of tumors primarily arises from factors such as aberrant expression of tumor‐associated antigens, an immunosuppressive microenvironment and epigenetic alterations. These factors work together to enable tumors to evade immune recognition, ultimately leading to the inability of the immune system to launch effective attacks against them. This property represents a fundamental mechanism of tumor immune evasion, critically influencing tumor progression and therapeutic efficacy. Moreover, it serves as a key factor of immunotherapy resistance and poor prognosis. Hence, it is of great clinical value to investigate efficient and low‐toxicity therapeutic strategies by enhancing melanoma immunogenicity.

Studies show that the induction of immunogenic cell death (ICD) restores systemic antitumor immune responses and improves the antitumor effects of immune checkpoint inhibitors. ICD is a specific form of programmed death that activates the innate immune response through the release of damage‐associated molecular patterns (DAMPs). In cancers, the absence of immunogenic stress signals such as DAMPs is detrimental to tumor prognosis. The DAMPs family contains critical immune mediators, including adenosine triphosphate (ATP) for chemotactic signaling, calreticulin (CRT) mediating phagocytosis, high‐mobility group box 1 (HMGB1) promoting dendritic cells (DCs) maturation, and type I interferon (IFN‐I) regulating inflammatory responses [3, 4]. DAMPs bind to antigen‐presenting cells (APCs), promoting dead cell antigens recognition, phagocytosis and presentation to effector T cells, thereby initiating adaptive immune responses [5]. The cGAS‐STING pathway synergizes with DAMPs to induce ICD in tumors. Under tumor stress, the release of mitochondrial DNA (mtDNA) serves as a key DAMPs event. This mtDNA, resembling the molecular patterns of microbial pathogens, activates the cGAS‐STING axis to trigger IFN‐I production [6, 7, 8]. This enhances APC‐mediated phagocytosis of dead cell antigens and CD8^+^ T‐cell activation. Melanoma studies demonstrate that mtDNA leakage activates cGAS‐STING signaling, driving IFN‐I‐dependent T‐cell infiltration and ICD initiation, highlighting its dual role in amplifying antitumor immunity through DAMP release and immune recognition [9].

The dried root of *Astragalus membranaceus* (Fabaceae) is used in traditional Chinese medicine to tonify *Qi*. It possesses notable antitumor, immunomodulatory, and other bioactivities, with clinical trials supporting its use in oncological regimens [10, 11, 12, 13, 14, 15, 16, 17]. *Astragalus* polysaccharides (APS) are the core active ingredient of *Astragalus membranaceus*, and modern pharmacological studies have shown that APS can exert antitumor effects by regulating intestinal microbes and influencing immunity [18, 19, 20, 21]. While the diversity of the intratumoral microbiota, which can be reshaped by the translocation of gut microbes, is closely linked to cancer patient prognosis, its mechanistic basis is not fully elucidated [22, 23]. The intratumoral microbiota plays a key role in shaping the local immune response to the tumor microenvironment. Research has also demonstrated that bacteria can enter melanoma cells and trigger an immune response in melanoma [22]. Another study found that microbiota‐associated metabolites can accumulate in murine tumors, activating the cGAS‐STING signaling and subsequently inducing ICD [24, 25]. Despite these insights, whether APS exerts its antitumor effects by remodeling the intratumoral microbiota to trigger this cGAS‐STING‐ICD cascade remains to be elucidated.

In this study, we demonstrate that APS inhibits melanoma growth by inducing ICD via the cGAS‐STING pathway. Leveraging 5R 16S rRNA sequencing, we identified *Pseudomonas* enrichment as a key driver, confirmed by the finding that *Pseudomonas* sterile supernatant is sufficient to suppress tumor growth. We propose that this activation arises not from a direct effect but as a transcriptional adaptive response to continuous microbial stimulation.

## 2 Materials and Methods

### 2.1 Isolation and Identification of APS

APS was isolated from *Astragalus membranaceus* by aqueous ethanol precipitation according to the reference [26]. Distilled water was used to isolate the APS, which was filtered and mixed with ethanol to a concentration of 70%. After three repeated precipitation processes, perform three rounds of protein removal on the crude polysaccharide using the Sevag method, and then freeze‐dry for subsequent experiments. Total sugar content of the obtained APS was quantified via the phenol‐sulfuric acid method using D‐glucose as the standard. Residual protein content in deproteinized APS was determined by the Bradford assay with bovine serum albumin (BSA) as the standard. Total sugar content and residual protein content across three independent batches are presented in Table S1, verifying the batch‐to‐batch consistency of APS. The monosaccharide composition of APS was analyzed by Gas Chromatography–Mass Spectrometry (GC–MS), performed by Prof. Wang Shunchun's team at Shanghai University of Traditional Chinese Medicine, following well‐established protocols [27]. GC–MS is shown in Figure S1. The APS used in this study consisted of four main monosaccharides: arabinose (16.21%), glucose (75.24%), galactose (6.76%), and rhamnose (1.79%).

### 2.2 Cell Lines and Cell Culture

Mouse melanoma cell line B16F10 was purchased from the Cell Bank of the Chinese Academy of Sciences (Shanghai, China). B16F10 cells were cultured in Roswell Park Memorial Institute (RPMI) 1640 Medium (31800022, Gibco, Thermo Fisher Scientific, USA) with 10% (v/v) fetal bovine serum (FBS, Gibco, Thermo Fisher Scientific, USA) and 1% penicillin/streptomycin (C0222, Beyotime, China) at 37°C China humidified atmosphere with 5% CO_2_. The cells were expanded and frozen in multiple vials after the 3rd generation and passaged in culture for ≤ 4 months after being thawed from authentic stocks. The culture medium was replaced every 2–3 days. Over 80% of confluent cells were subcultured by splitting them at 1:3 ratios. Cell lines were routinely tested for mycoplasma contamination.

### 2.3 Mice and Tumor Models

C57BL/6 mice (male, 6–8 weeks old, 18–20 g) were obtained from Shanghai SLAC Laboratory Animal Co. Ltd. (Shanghai, China). All mice were housed under specific pathogen‐free (SPF) conditions in a controlled environment with ad libitum access to standard rodent chow and sterile water. B16F10 cells in logarithmic growth phase were taken and digested. Cell suspension was collected using a 1 mL sterile syringe, and injected subcutaneously into the right axilla of C57BL/6 mice, at 2 × 10^5^ per mouse. The modeling day was designated as Day 0, and the mice were randomly grouped for treatment on the 6th day after tumor inoculation.

### 2.4 Grouping and APS Administration

#### 2.4.1 APS Efficacy

On Day 6 posttumor inoculation, mice were randomized into four groups (*n* = 6/group): Model, APS‐low (100 mg/kg), APS‐medium (200 mg/kg), and APS‐high (400 mg/kg). From Day 7, APS groups received oral APS in sterile water daily for 13 days; the Model group received sterile water only. All procedures were performed under SPF conditions using sterile solutions and individual gavage needles.

#### 2.4.2 Mice Survival Time

The modeling and drug administration method is the same as the “APS efficacy” (*n* = 10/group). In this experiment, the mice were treated with APS until they died naturally or the tumor volume reached the ethical criteria for death (unilateral tumor diameter ≥ 2 cm).

#### 2.4.3 cGAS Inhibitor Intervention

There were four groups: Model, APS, RU.521 (cGAS inhibitor, M9447, AbMole, China), APS + RU.521 group (*n* = 6/group). The APS group and APS + RU.521 group were given APS‐200 mg/kg by gavage starting from the 7th day after modeling. Model and RU.521 groups were given an equal amount of water once daily. The RU.521 group and the APS + RU.521 group were injected intraperitoneally with RU.521 (5 mg/kg) solution daily. The Model and APS groups were injected with an equal amount of saline intraperitoneally daily for 13 days.

#### 2.4.4 Intratumoral Antibiotic Cocktail Injection

There were four groups: Model, APS, Antibiotic cocktail (ABX), and APS + ABX group (*n* = 6/group). 200 mg/kg of APS was given to the APS group and APS + ABX group by gavage from the 7th day after modeling; the same amount of water was given to the Model group and ABX group once/day. The ABX and APS + ABX group were injected intratumorally with ABX solution every other day, and a mixture of antibiotics was prepared using sterilized ultrapure water, as the concentrations of ampicillin, neomycin, and metronidazole were 1 g/L, and the concentration of vancomycin was 500 mg/L. This was done for 13 days.

#### 2.4.5 Microbiota Rescue Using Sterile Supernatants From *Pseudomonas*

There were four groups: Antibiotic cocktail (ABX), ABX+ *Pseudomonas* sterile supernatants (ABX + *P*.), ABX + APS (ABX + APS), ABX + APS + *P*. group (*n* = 6/group). To deplete indigenous microbiota, all mice received the antibiotic cocktail for 7 days prior to tumor inoculation. On the day of injection, cell suspensions in the ABX + *P*. and ABX + APS + *P*. groups were resuspended in *Pseudomonas* sterile supernatant mixture; in contrast, cell suspensions in the ABX and ABX + APS groups were resuspended in sterile PBS. All cell suspensions were then subcutaneously injected into the axillary region of mice. Starting on Day 7 postinoculation, APS was administered 200 mg/kg via oral gavage daily for 13 days, while the other groups received an equivalent volume of sterile water.

### 2.5 Hematoxylin–Eosin Staining

Fresh tumor tissues were collected, soaked and fixed in 4% paraformaldehyde (30525‐89‐4, Titans, China) for more than 24 h for paraffin‐embedded sections. Tumor tissue sections were baked at 60°C for 1 h, deparaffinized in xylene, and rehydrated through a graded ethanol series. The sections were stained with hematoxylin and eosin. After sealing the sections, the morphology of the tissue was examined with a microscope (Japan, Olympus).

### 2.6 Immunofluorescence

Paraffin‐embedded tumor sections were baked at 60°C for 1 h, dewaxed, and rehydrated. After antigen retrieval, sections were permeabilized with PBS containing 1% Triton X‐100 (P0096, Beyotime) and blocked with 5% BSA (4240GR025, BioFroxx). The primary antibodies were incubated overnight at 4°C, and the secondary antibodies were incubated at room temperature. Nuclei were counterstained with DAPI (2 μg/mL, C0065, Solarbio). Images were acquired using an Olympus microscope. For the list of antibodies used, please refer to Table 1.

**TABLE 1:** Antibodies used in immunofluorescence.

Antibodies	Dilution	Identifier	Source
CRT	1:500	27298–1‐AP	Proteintech, USA
HMGB1	1:500	10829–1‐AP	Proteintech, USA
*E. coli* LPS	1:500	ab35654	Abcam, USA
LTA	1:200	HM20a48	Hycult Biotech, USA
CoraLite488‐conjugated goat anti‐rabbit IgG(H + L)	1:1000	SA00013‐2	Proteintech, USA
CoraLite488‐conjugated goat anti‐mouse IgG(H + L)	1:1000	SA00013‐1	Proteintech, USA

### 2.7 Flow Cytometry

Tumor tissues were dissociated with Collagenase Type IV 1 mg/mL (LS004188, Worthington Biochemical, USA), Neutral protease 1 mg/mL (88922N, Worthington Biochemical, China), and Deoxyribonuclease I 0.02 mg/mL (LS002139, Worthington Biochemical, USA). Tumor draining lymph nodes (TDLNs) were placed into a six‐well plate containing RPMI 1640 complete culture medium and fully ground using a sterile syringe. The 70 μm filter was used to obtain a single‐cell suspension. Erythrocyte was removed by Red Blood Cell Lysis Buffer (C3702, Beyotime, China). The cells were then incubated in PBS buffer supplemented with 1% FBS and fluorescent antibodies for 15 min for staining, determined by BD FACSVerse Flow cytometer. FlowJo_V10 software was used for data analysis. Please see Table 2 for a complete list of antibodies used in this study.

**TABLE 2:** Fluorescent antibodies used in this study.

Antibodies	Fluorescent dye	Identifier	Source
Fixable viability stain 780	APC‐Cy7	565388	BD Pharmingen, USA
FC Block	—	5502714	BD Pharmingen, USA
CD45	FITC	553079	BD Pharmingen, USA
CD45	PE‐Cy7	25‐0451‐81	Invitrogen, USA
CD3	PerCP.Cy5.5	551163	BD Pharmingen, USA
CD4	FITC	553046	BD Pharmingen, USA
CD8	PE	553032	BD Pharmingen, USA
MHC‐II	PE	557000	BD Pharmingen, USA
CD11c	APC	550261	BD Pharmingen, USA
CD80	PerCP.Cy5.5	560526	BD Pharmingen, USA
CD86	PE‐Cy7	560582	BD Pharmingen, USA

### 2.8 Enzyme‐Linked Immunosorbent Assay (ELISA) for HMGB1 and Annexin A1 (ANXA1) Expression Levels

Blood samples were collected and allowed to clot at 4°C for 2 h, followed by centrifugation at 3000 × g for 20 min (4°C) to obtain serum. Serum levels of HMGB1 (EM30645M, Biotechwell, China) and ANXA1 (ml374057, Mlbio, China) were quantified using commercial ELISA kits according to the manufacturers' protocols.

### 2.9 ATP Assay

Tumor tissues were collected and homogenized in lysis buffer (100 μL per 20 mg tissue) using an ATP assay kit (S0026, Beyotime, China). After complete lysis, samples were centrifuged at 12,000 × g for 5 min at 4°C. The supernatant was collected, and ATP levels were quantified by measuring relative luminescence units (RLU) using a chemiluminescence meter.

### 2.10 Intratumoral Microbial Diversity Detection

Prior to tumor tissue collection, the surgical area was sequentially disinfected with povidone‐iodine solution (10%) followed by 75% ethanol sterilization. Genomic DNA was extracted using a commercial kit, followed by multiplex PCR amplification targeting five hypervariable regions of the 16S rRNA gene. The amplified products were sequenced using 5R 16S sequencing technology. Microbial taxonomic classification was performed using the Short Multiple Regions Framework (SMRF) algorithm, followed by α‐ and β‐diversity analyses and differential abundance testing.

### 2.11 Total RNA Extraction and Real‐Time Quantitative Polymerase Chain Reaction (RT‐qPCR)

Total RNA from cells or tumor tissues was extracted using the EZB RNA Purification Kit (B004DP, EZBioscience, USA). RNA concentrations were determined by nanodrop and then normalized. cDNA was synthesized from 1 μg of total RNA using the EZB Reverse Transcription Kit (A0010GQ, EZBioscience, USA). qPCR reactions were performed using SYBR Green kit (A0001‐R2, EZBioscience, USA) on an ABI Q7 Flex real‐time PCR system (QuantStudio 7 Flex, Thermo Fisher, USA). The data were quantified using the 2^−ΔΔCt^ method. GAPDH was used as an internal control for quantification. Please see Table 3 for a complete list of primers used in this study.

**TABLE 3:** Primer sequences used in this study.

Primer	Primer sequence
*Ifnα* forward	5′‐GGA TGT GAC CTT CCT CAG ACT C‐3′
*Ifnα* reverse	5′‐ACC TTT CTC CTG CGG GAA TCC AA‐3′
*Ifnβ* forward	5′‐ATG ACC AAC AAG TGT CTC CTC C‐3′
*Ifnβ* reverse	5′‐GGA ATC CAA GCA AGT TGT AGC TC‐3′
*Gapdh* forward	5′‐AGG TCG GTG TGA ACG GAT TTG‐3′
*Gapdh* reverse	5′‐TGT AGA CCA TGT AGT TGA GGT CA‐3′

### 2.12 Western Blotting

Cells or tumor tissues were lysed in RIPA buffer (P0013D, Beyotime, China) containing 1 mM phenylmethylsulfonyl fluoride, protease inhibitor and phosphatase inhibitor cocktail (WB0122, Biotech well, China). Lysates were spun at 10000 × *g* for 10 min and the supernatants kept at −20°C until use. Proteins were separated by SDS‐PAGE and transferred to nitrocellulose filter membranes; blots were probed with appropriate combinations of primary and HRP‐conjugated secondary antibodies. For repeated immunoblotting, membranes were stripped in Western Blot Stripping Buffer for 10 min at room temperature prior to reprobing (WB6200, NCM Biotech, China). The antibody used in this study were displayed in Table 4.

**TABLE 4:** Antibodies used in western blotting.

Antibodies	Dilution	Identifier	Source
TBK1	1:1000	T55145	Abmart, China
Phospho‐TBK1	1:1000	T58364	Abmart, China
Iκκα/β	1:1000	TP56290	Abmart, China
Phospho‐Iκκα/β	1:1000	T55660	Abmart, China
IRF3	1:1000	T55779	Abmart, China
Phospho‐IRF3	1:1000	TA2436	Abmart, China
NF‐κB	1:1000	8242	CST, USA
Phospho‐NF‐κB	1:1000	3033	CST, USA
STING	1:1000	A21051	Abclonal, China
Phospho‐STING	1:1000	AP1199	Abclonal, China
cGAS	1:1000	A23846	Abclonal, China
GAPDH	1:20000	60,004–1‐Ig	Proteintech, USA
Anti‐mouse IgG‐HRP	1:20000	BL001A	Biosharp, China
Anti‐rabbit IgG‐HRP	1:20000	BL003A	Biosharp, China

### 2.13 Isolation of Sterile Supernatant Mixture From Three *Pseudomonas* Species

Based on the literature [28], lyophilized *Pseudomonas* strains ( *P. moraviensis* , bio02504, Titans; *P. aeruginosa* , ATCC 9027; *P. stutzeri* , CICC 10402, Beijingbio) were cultured in Tryptic Soy Agar (TSA, HBKP0177, Hopebiol) at 150 rpm for 48 h. Bacterial suspensions (2 × 10^9^ CFU/mL) were centrifuged at 3000 g for 10 min, and the supernatants were filtered through 0.22 μm nitrocellulose filter. Filtered supernatants were diluted in culture medium to 10%, 15%, and 20% (v/v) for subsequent experiments [29].

### 2.14 Methylthiazolyldiphenyl‐Tetrazolium Bromide (MTT) Assay

After sterile supernatant mixture treatment, the cells were incubated with MTT solution (5 mg/mL, ST316, Beyotime, China) at 37°C for 4 h. MTT crystals were dissolved with dimethyl sulfoxide (DMSO), and the absorbance was measured by an enzyme meter at 490 nm.

### 2.15 Apoptosis Assay

Cells were inoculated in 6‐well plates, treated with sterile supernatant mixture from three *Pseudomonas* species, culture supernatant was collected and cells were washed with PBS. Cells were then stained using FITC Annexin V Apoptosis Detection Kit I (556547, BD Pharmingen, USA) according to the manufacturer's protocol. Cells were detected using flow cytometry and the proportion of dead cells was assessed by counting early and late apoptosis. Flow Jo software was used to analyze the data.

### 2.16 Statistical Analysis

Data are presented as means ± SEM GraphPad Prism 10 software (GraphPad Software, USA) was utilized for the statistical analyses. Two‐tailed, unpaired Student's *t*‐tests were used to determine statistical significance between two groups. To check for normality, the Shapiro–Wilk test was conducted, if not met, a Mann–Whitney test was applied. In case of unequal variances (*F*‐test), the *t*‐test was adjusted using Welch's correction. For comparisons of more than two groups, one‐way or two‐way ANOVA with Tukey's post hoc test was used. *p* values < 0.05 were considered statistically significant.

## 3 Result

### 3.1 Antitumor Efficacy and Safety of APS in Melanoma

**FIGURE 1:**
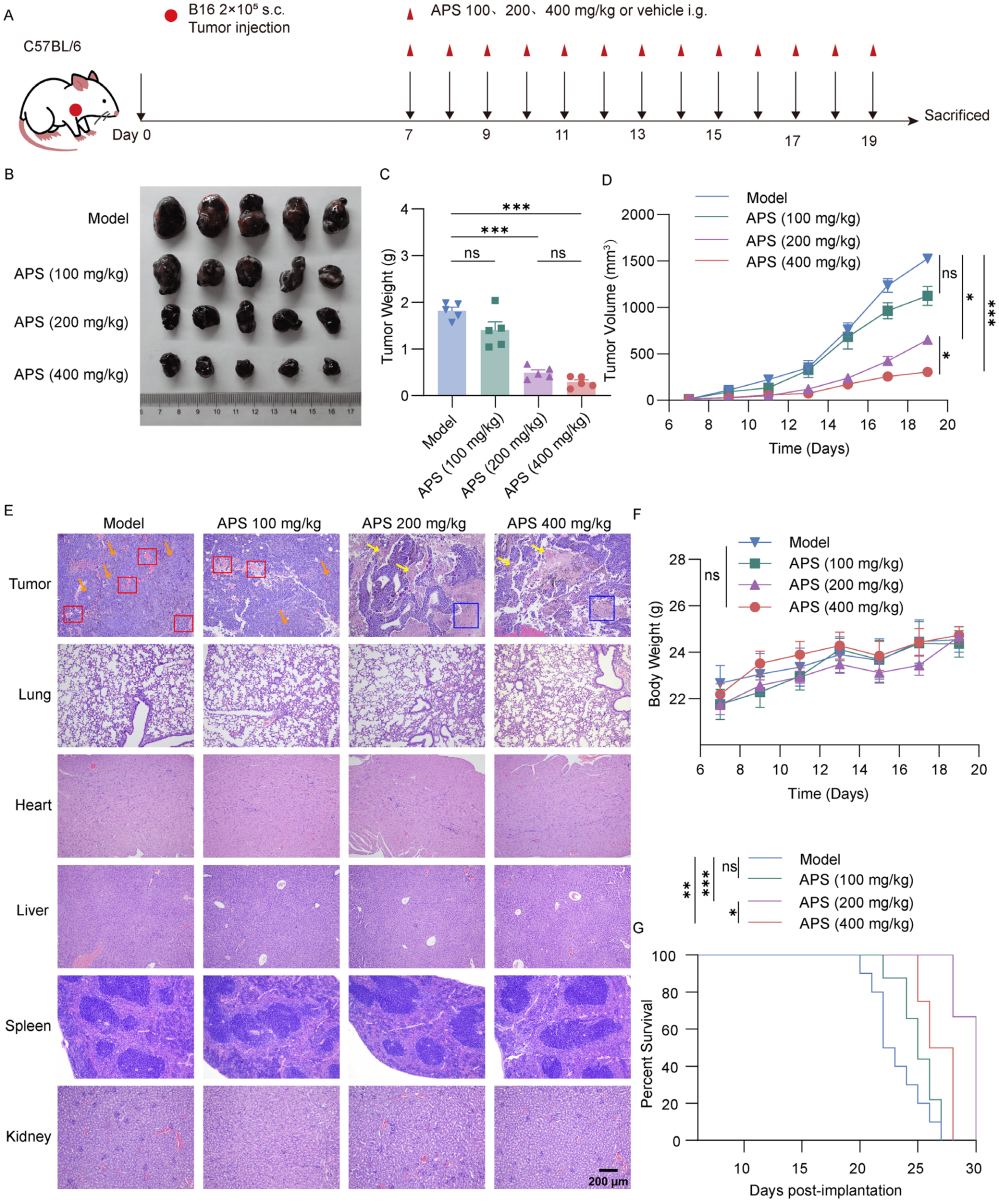
APS inhibits melanoma growth and prolongs survival in mice. (A) In vivo experimental design. (B, C) Representative tumor images and weights collected at 19 days postinoculation (*n* = 5). (D) Tumor volume measurements during the experimental period. (E) H&E staining of tumor tissues and major organs (lung, heart, liver, spleen, kidney; scale bar: 200 μm, *n* = 3; Red box: Vascularity; Orange arrow: Mitotic; Blue box: Coagulative necrosis; Yellow arrow: Nuclear pyknosis and eosinophilic cytoplasm). (F) Body weight changes across treatment groups. (G) Survival curves of model and APS‐treated groups (*n* = 10). Data were presented as mean ± SEM. NS, not significant; *p < 0.05, **p < 0.01, ***p < 0.001.

APS treatment (100, 200, and 400 mg/kg) was initiated on Day 7 post‐B16F10 cell inoculation (Figure 1A). APS produced dose‐dependent antitumor effects, progressively reducing tumor volume and mass (Figure 1B–D). Histopathology showed high vascularity and mitosis in the model group, while APS groups exhibited coagulative necrosis with nuclear pyknosis and eosinophilic cytoplasm. Major organs showed no significant damage (Figure 1E), and body weight remained stable, indicating low toxicity (Figure 1F). Survival was optimal in the APS‐200 mg/kg group, with 50% of mice surviving beyond Day 28; the 400 mg/kg group had slightly shorter survival (Figure 1G). These findings collectively demonstrate that APS possesses antitumor activity with an acceptable safety profile in melanoma mice. Specifically, antitumor efficacy improved with increasing doses from 100 mg/kg to 200 mg/kg, but higher doses (400 mg/kg) failed to enhance efficacy and even compromised survival, likely owing to immune overactivation. Thus, APS 200 mg/kg was identified as the optimal dose for subsequent investigations, balancing maximal antitumor activity and minimal adverse effects.

### 3.2 APS Induced ICD and Modulated the Tumor Immune Microenvironment in Melanoma Mice

**FIGURE 2:**
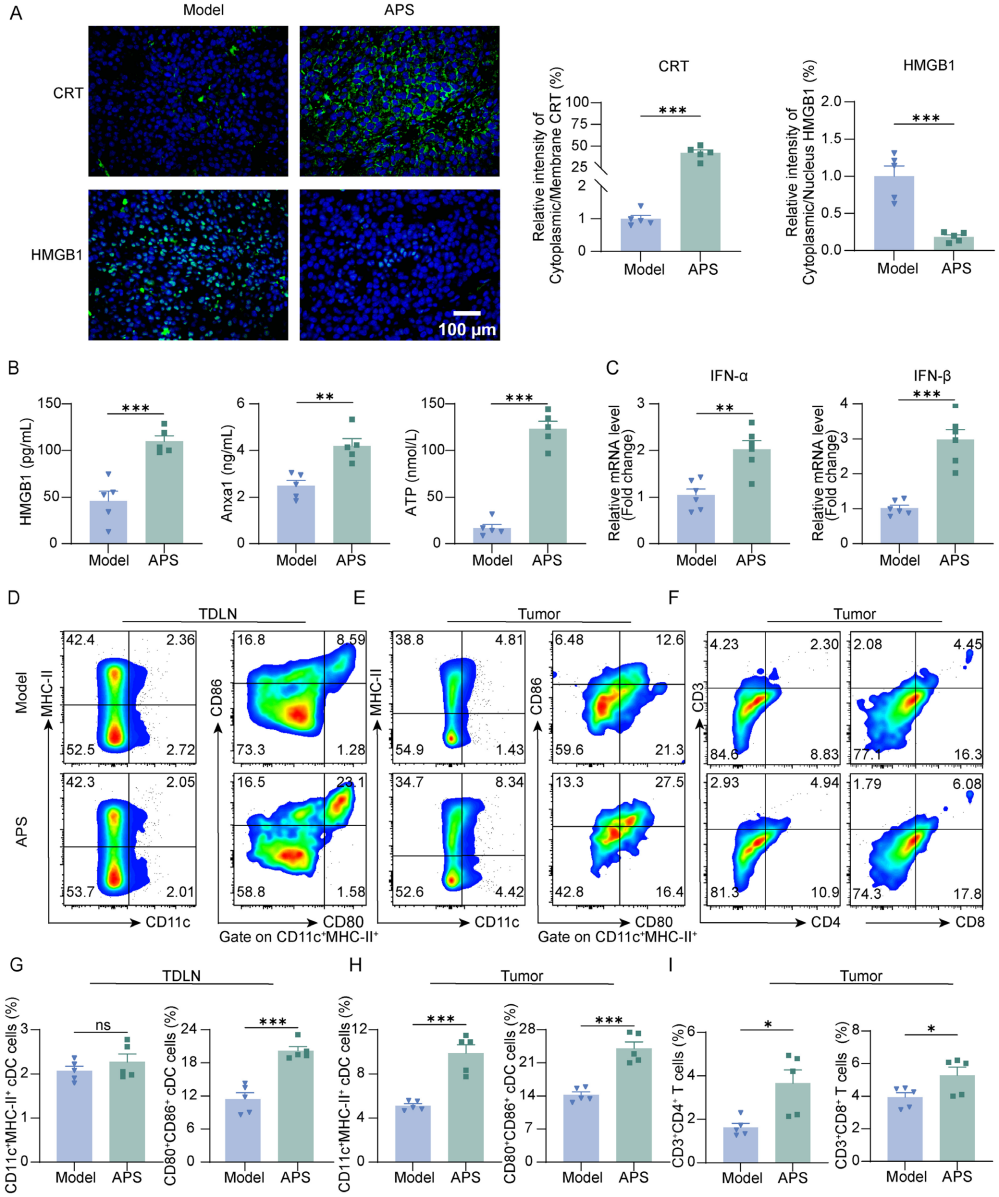
APS induced ICD and modulated the tumor immune microenvironment in melanoma mice. (A) Immunofluorescence analysis and quantification of CRT and HMGB1 expression in tumor tissues (scale bar: 100 μm). (B) Serum levels of HMGB1 and ANXA1, along with intratumoral ATP content. (C) IFN‐α and IFN‐β mRNA expression in tumor tissues measured by RT‐qPCR. (D, E) Flow cytometric analysis of MHC‐II^+^CD11c^+^ DCs and CD80^+^CD86^+^ DCs in TDLNs and tumor tissues. (F) Tumor‐infiltrating CD4^+^ and CD8^+^ T‐cell populations analyzed by flow cytometry. (G–I) Statistic analysis of MHC‐II^+^CD11c^+^ DCs, CD80^+^CD86^+^ DCs, CD4^+^ T cells, and CD8^+^ T cells. Data were presented as mean ± SEM (*n* = 5–6). ns, not significant; *p < 0.05, **p < 0.01, ***p < 0.001.

Immune evasion due to impaired tumor immunogenicity is a key mechanism in cancer progression. Induction of ICD has been shown to counteract immunosuppression and activate systemic antitumor immunity [30]. Given the known immunomodulatory effects of APS [31, 32, 33], we examined its potential to induce ICD in melanoma. Key ICD markers—including CRT membrane translocation, IFN‐α/β expression, ATP release, and serum levels of ANXA1 and HMGB1—were assessed. Immunofluorescence analysis revealed pronounced exposure of CRT on tumor tissues following APS treatment (Figure 2A). Additionally, it was observed that HMGB1 transferred from the nucleus to the cytoplasm (Figure 2A), accompanied by elevated levels of ANXA1 and HMGB1 in the serum (Figure 2B). APS also increased the expression of ATP and IFN‐α/β mRNA in tumor tissues (Figure 2B,C), confirming APS‐induced ICD. The tumor antigens and danger signals released by ICD can enable APCs to process tumor antigens and present them via MHC‐I/II, with concomitant upregulation of CD80/CD86, leading to T‐cell activation and immune memory [34]. In TDLNs and tumor tissues, flow cytometry revealed expanded CD11c^+^MHC‐II^+^ DCs populations with higher CD80/CD86 expression following APS treatment (Figure 2D,E,G,H). APS also increased infiltration of CD4^+^ and CD8^+^ T cells in tumors (Figure 2F,I). These results indicate that APS triggers ICD, promotes DC maturation in TDLNs and tumor sites, and enhances T cell‐mediated antitumor immunity.

### 3.3 APS Activated the cGAS‐STING Pathway to Induce ICD in Melanoma Mice

**FIGURE 3:**
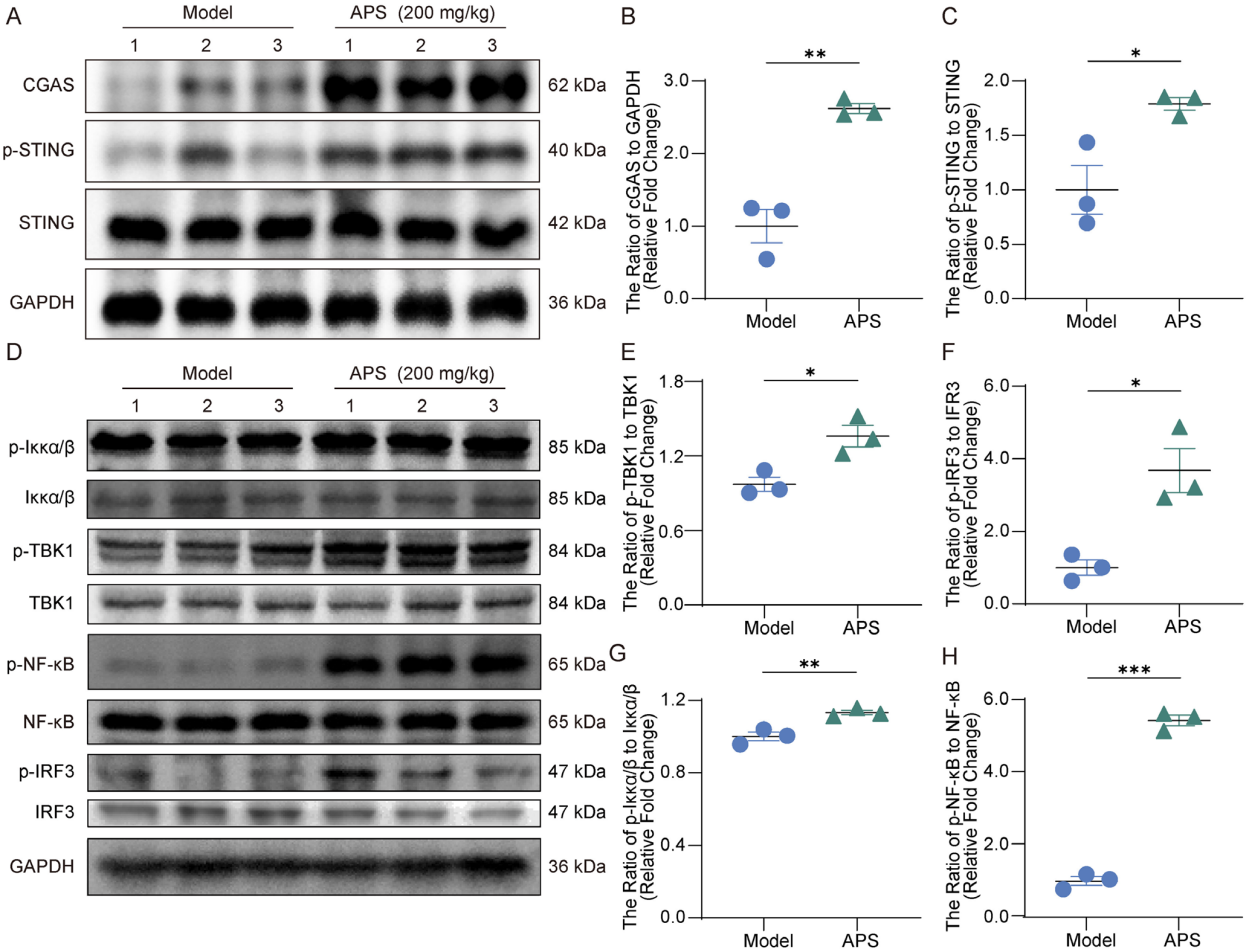
APS activated the cGAS‐STING signaling axis in melanoma mice. (A–C) Western blot analysis of cGAS, STING, and phosphorylated‐STING (p‐STING) protein expression in tumor tissues. (D–H) Western blot detection of total and phosphorylated protein levels of IKKα/β, TBK1, IRF3, and NF‐κB in tumor tissues. Data were presented as mean ± SEM (*n* = 3). *p < 0.05, **p < 0.01, ***p < 0.001.

**FIGURE 4:**
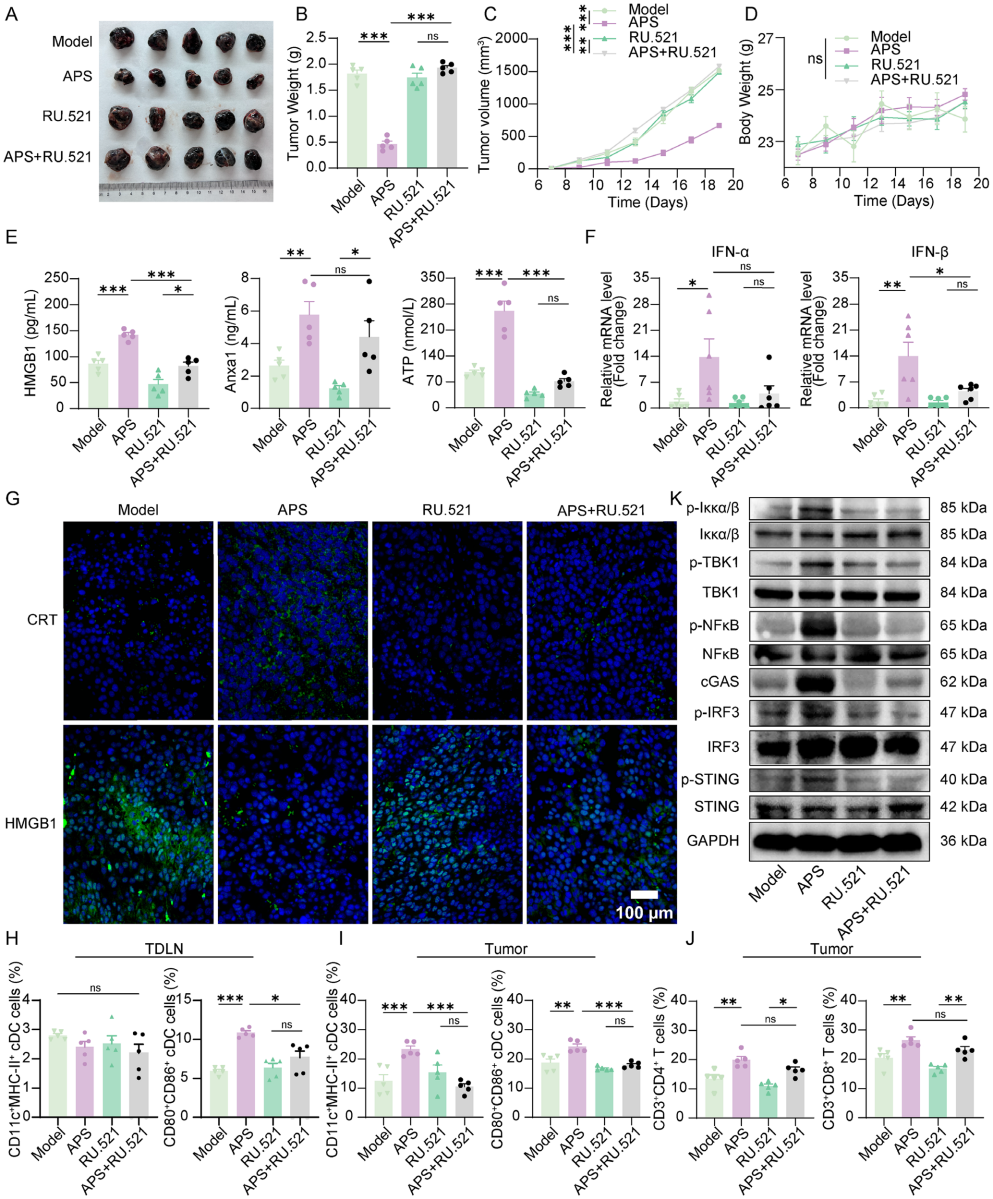
APS‐induced ICD and antitumor activity depended on intact cGAS‐STING signaling. (A, B) Representative tumor tissue images and corresponding weights collected at 19 days postinoculation. (C) Tumor volume measurements recorded during the experimental period. (D) Body weight changes across experimental groups. (E) Serum levels of ANXA1 and HMGB1, along with intratumoral ATP content. (F) IFN‐α and IFN‐β mRNA expression in tumor tissues measured by RT‐qPCR. (G) Immunofluorescence analysis of CRT and HMGB1 expression in tumor sections (scale bar: 100 μm). (H, I) Flow cytometric analysis of MHC‐II^+^CD11c^+^ DCs and CD80^+^CD86^+^ DCs in TDLNs and tumor tissues. (J) Flow cytometric analysis of CD4^+^ and CD8^+^ T‐cell infiltration in tumor tissues. (K) Western blot detection of cGAS‐STING pathway proteins in melanoma tissues. Data were presented as mean ± SEM (*n* = 3–6). ns, not significant; *p < 0.05, **p < 0.01, ***p < 0.001.

Activation of the cGAS–STING pathway contributes to antitumor immunity by inducing ICD and remodeling the tumor microenvironment [6, 7]. In melanoma, this pathway enhances ICD and facilitates cytotoxic T lymphocyte‐mediated tumor clearance [35]. To determine whether APS modulates cGAS–STING signaling, we performed Western blot analysis on tumor tissues. APS treatment significantly increased cGAS expression and STING phosphorylation (Figure 3A–C), and promoted phosphorylation of downstream molecules TBK1, IRF3, and NF‐κB, along with activation of the IKKα/β complex (Figure 3D–H). To further investigate the functional role of this pathway in APS‐induced ICD, we used the cGAS inhibitor RU.521. Compared with the model group, the use of RU.521 alone had no significant effect on tumor growth or ICD marker expression (Figure 4A–D). However, co‐administration of RU.521 with APS markedly attenuated the antitumor efficacy of APS (Figure 4A–D). The activation of certain ICD markers (CRT, ATP, and IFN‐β) and nuclear‐to‐cytoplasmic HMGB1 translocation were weakened (Figure 4E–G). In contrast, Anxa1 serum levels and IFN‐α expression remained unchanged relative to APS monotherapy (Figure 4F). DC maturation markers were downregulated in the combination group (Figure 4H,I), and a decreasing trend in CD4^+^ and CD8^+^ T‐cell infiltration was observed, though not statistically significant (Figure 4J). Western blot confirmed that RU.521 inhibited APS‐induced activation of the cGAS–STING pathway (Figure 4K). Together, these results indicate that APS‐induced ICD and antitumor activity are partially dependent on functional cGAS–STING signaling.

### 3.4 Depletion of Intratumoral Microbiota Partially Attenuated APS‐Induced ICD

**FIGURE 5:**
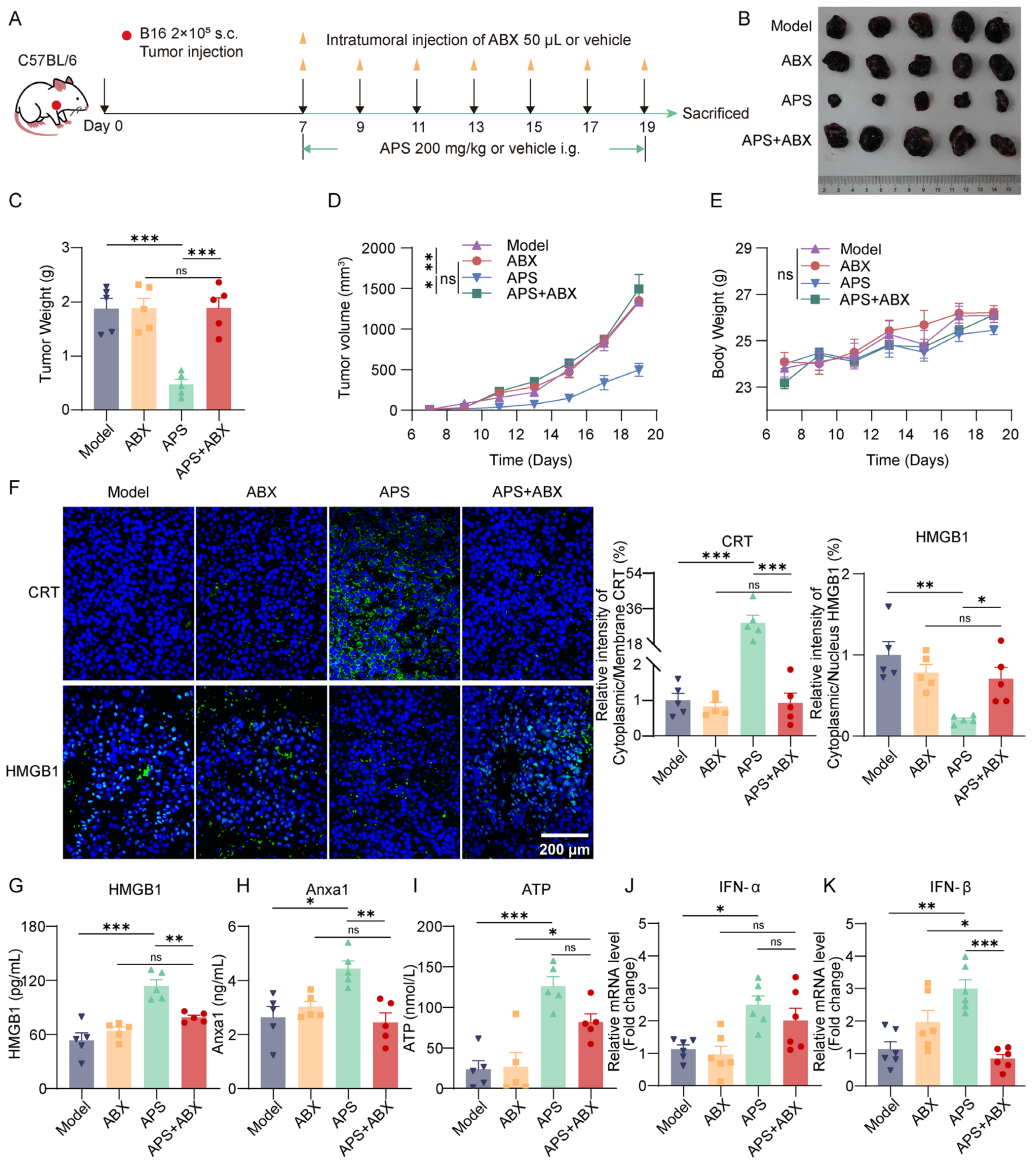
Depletion of intratumoral microbiota attenuated APS‐mediated antitumor effects and ICD. (A) In vivo experimental design. (B, C) Representative tumor images and weights collected at 19 days postinoculation. (D) Tumor volume measurements during treatment. (E) Body weight changes across groups. (F) Immunofluorescence staining of CRT and HMGB1 in tumor tissues (scale bar: 200 μm). (G, H) Serum levels of HMGB1 and Anxa1. (I) Intratumoral ATP content. (J–K) IFN‐α and IFN‐β mRNA expression in tumor tissues. Data were presented as mean ± SEM (*n* = 5–6). ns, not significant; *p < 0.05, **p < 0.01, ***p < 0.001.

**FIGURE 6:**
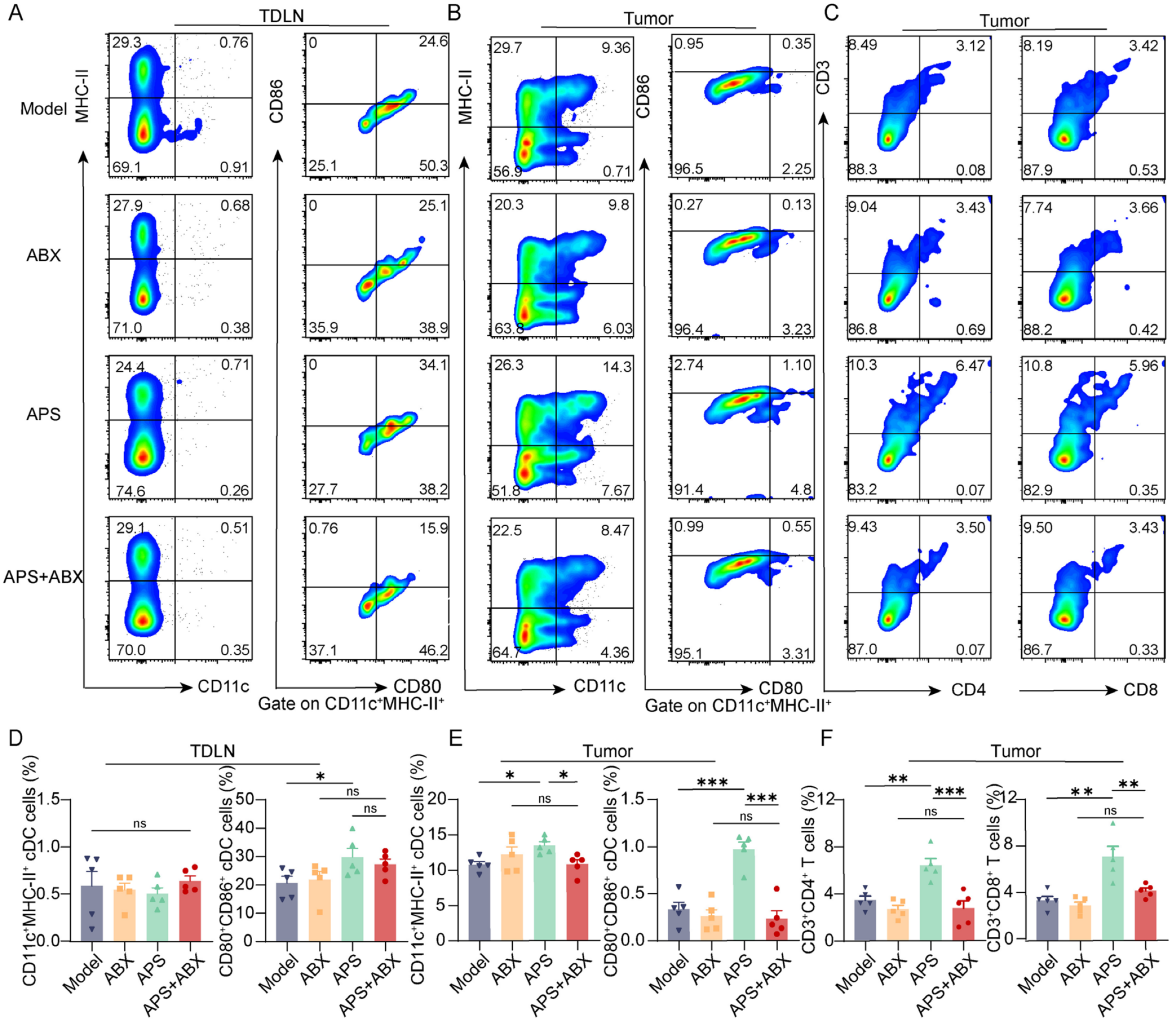
Depletion of intratumoral microbiota diminished APS‐mediated modulation of the tumor immune microenvironment in melanoma‐bearing mice. (A, B) Flow cytometric analysis of MHC‐II^+^CD11c^+^ DCs and CD80^+^CD86^+^ DCs in TDLNs and tumor tissues. (C) Flow cytometric detection of CD4^+^ T cells and CD8^+^ T cells in tumor tissues. (D–F) Flow cytometric analysis of CD4^+^ T cells, CD8^+^ T cells, MHC‐II^+^CD11c^+^ DCs, and CD80^+^CD86^+^ DCs. Data were presented as mean ± SEM (*n* = 5). ns, not significant; *p < 0.05, **p < 0.01, ***p < 0.001.

Prior studies have shown that intratumoral microbiota exert antitumor effects by activating STING signaling to promote CD8^+^ T‐cell infiltration [25]. Notably, these intratumoral microbiota originate from the gut. Our Previous work demonstrated that APS modulates the gut microbiota in melanoma‐bearing mice. Herein, we further investigated whether APS exerts antitumor effects by regulating intratumoral microbiota. We administered ABX via intratumoral injection on Day 7 posttumor inoculation to eliminate intratumoral bacteria in melanoma mice, followed by APS treatment (Figure 5A). Immunofluorescence detection of LPS (a major component of Gram‐negative bacterial outer membrane) and LTA (a key constituent of Gram‐positive bacterial cell walls) confirmed effective bacterial clearance in tumor tissues following ABX treatment (Figure S2A). Combined ABX + APS treatment attenuated the tumor growth inhibition observed with APS monotherapy (Figure 5B–D), while no significant differences in body weight were detected among groups (Figure 5E), suggesting microbiota‐dependent mechanisms in APS antitumor activity. Notably, ABX co‐treatment significantly reduced APS‐induced ICD marker activation, including CRT surface exposure, HMGB1 and Anxa1 release, and IFN‐β mRNA expression (Figure 5F–H,K), though ATP release and IFN‐α mRNA expression remained unaffected (Figure 5I,J). Flow cytometry showed that immune cell infiltration was reduced in ABX + APS‐treated tumors compared with APS. Specifically, CD11c^+^MHC‐II^+^ DCs decreased, CD80/CD86 expression declined, and CD4^+^, CD8^+^ T‐cell proportions dropped (Figure 6B,C,E,F). However, TDLNs showed no significant changes (Figure 6A,D). These findings collectively demonstrate that APS‐triggered ICD and subsequent immune microenvironment remodeling may be partially mediated by intratumoral microbiota.

### 3.5 Depletion of Intratumoral Microbiota Attenuated APS‐Mediated Activation of the cGAS‐STING Signaling Pathway

**FIGURE 7:**
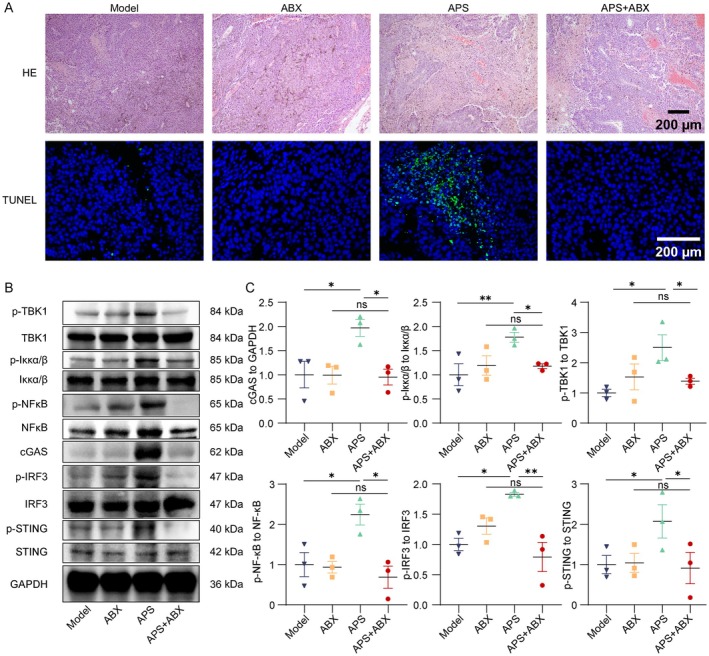
Microbial depletion attenuated APS‐induced activation of the cGAS‐STING signaling pathway. (A) H&E and TUNEL staining of tumor tissues across experimental groups (scale bar: 200 μm). (B) Western blot analysis of cGAS‐STING pathway proteins in tumor tissues. (C) Quantitative analysis of cGAS‐STING pathway protein expression. Data were presented as mean ± SEM (*n* = 3). ns, not significant; *p < 0.05, **p < 0.01, ***p < 0.001.

We further investigated the effect of intratumoral microbiota depletion on APS‐induced tumor cell apoptosis and cGAS‐STING pathway activation, to clarify whether microbiota participate in APS‐mediated antitumor immunity by regulating this pathway. Histological analysis using H&E and TUNEL staining revealed that microbial depletion attenuated APS‐induced tumor necrosis and apoptosis (Figure 7A). To investigate whether antibiotic‐mediated elimination of intratumoral microbiota would impair APS activation of cGAS‐STING signaling, we performed Western blot analysis. ABX treatment suppressed APS‐induced upregulation of cGAS expression and reduced the levels of p‐STING along with diminished activation of downstream signaling molecules including p‐NF‐κB, p‐TBK1, p‐IRF3, and p‐Iκκα/β (Figure 7B,C). These results demonstrate that intratumoral microbiota serve as essential mediators for APS‐mediated cGAS‐STING pathway activation and subsequent ICD.

### 3.6 APS‐Modulated Intratumoral Microbiota Composition in Melanoma Mice

**FIGURE 8:**
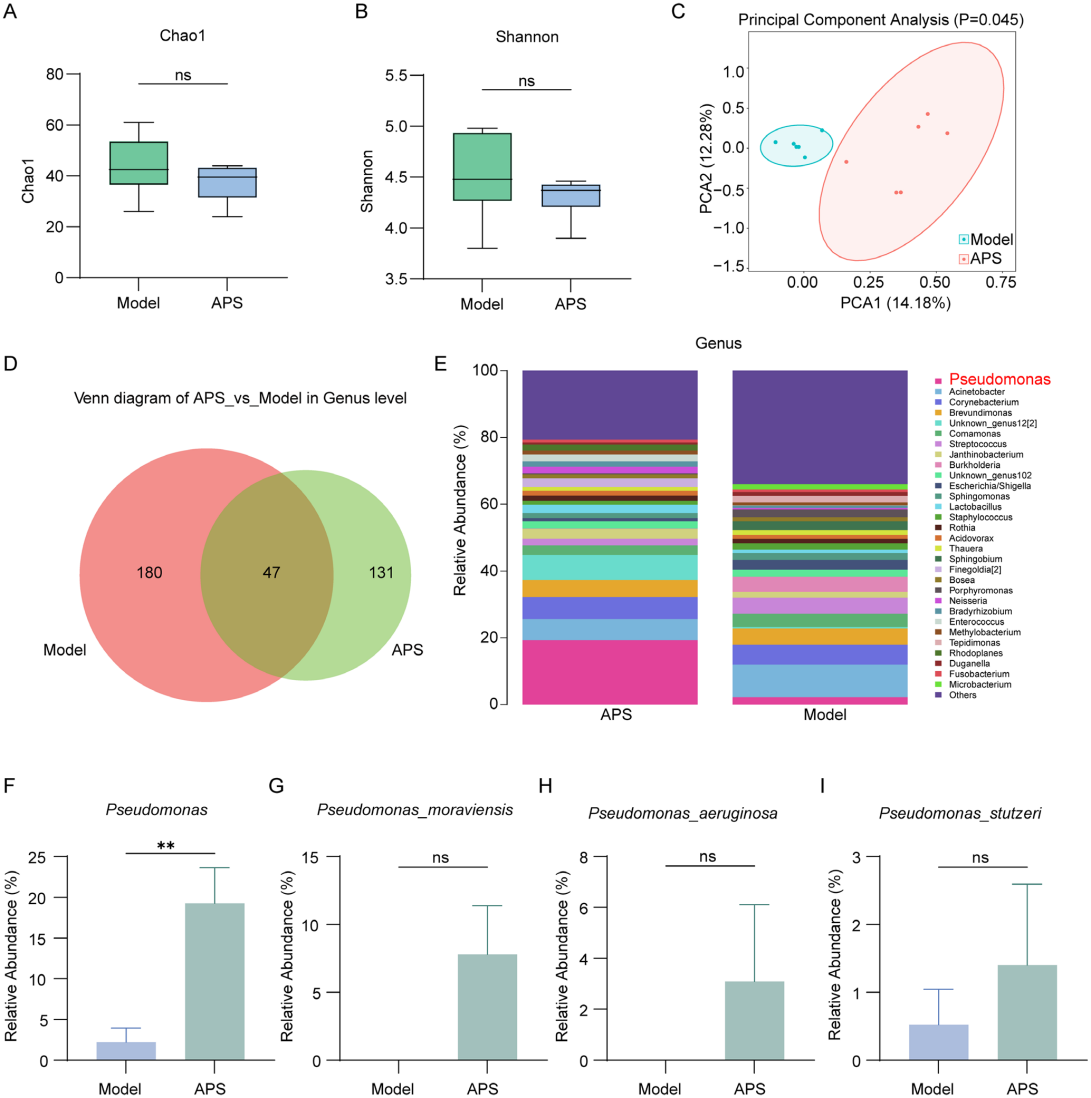
APS modulated intratumoral microbiota composition in melanoma mice. (A) Chao1 index analysis of α‐diversity in intratumoral microbiota. (B) Shannon index analysis of α‐diversity in intratumoral microbiota. (C) PCA of microbial composition between model and APS‐treated groups. (D) Venn diagram illustrating differential microbial composition between groups. (E) Stacked bar chart showing relative abundance of dominant bacterial genera. (F) Comparative analysis of *Pseudomonas* composition at genus level between model and APS‐treated groups. (G–I) Comparative analysis of specific microbial species between experimental groups. ns, not significant; **p < 0.01.

Previous studies from our team demonstrated that APS exerted antitumor effects through regulation of the gut microbiota‐immune axis [36]. Considering that gut microbiota can translocate and colonize tumor sites via the gut‐tumor axis to activate cGAS‐STING signaling and promote antitumor immune responses [23], we further investigated APS‐induced alterations in intratumoral microbial composition. Maintain strict aseptic technique during all sampling and administration procedures. APS changed the microbial composition in tumor; however, 5R 16S rRNA sequencing analysis revealed no significant differences in α‐diversity (Chao and Shannon indices) between APS and model groups (Figure 8A,B). This could be attributed to APS‐induced restructuring of the bacterial community: the abundance of dominant genera shifted dynamically, whereas the overall species richness and evenness remained unchanged. Therefore, no significant fluctuations in α‐diversity were observed. Principal component analysis (PCA) demonstrated distinct microbial community distributions between groups (Figure 8C). Venn diagram analysis identified 227 and 131 unique operational taxonomic units (OTUs) in model and APS groups respectively, with 47 shared OTUs (Figure 8D). At the genus level, APS treatment significantly reduced the relative abundance of *Corynebacterium*, *Brevundimonas*, *Comamonas*, *Streptococcus*, and *Burkholderia* while increasing *Pseudomonas*, *Acinetobacter*, and *Janthinobacterium* (Figure 8E). Although APS altered the abundances of the bacterial genera, only the abundance change of *Pseudomonas* was statistically significant at the genus level (Figure 8F, Figure S3). No *Pseudomonas* was detected in the blank control group, confirming no exogenous contamination in the experimental environment. The relative abundance of *Pseudomonas* was extremely low in the Model group (mean 2%; some samples were below the detection limit). After APS treatment, this value increased significantly to 19.25%, making *Pseudomonas* one of the dominant bacterial genera.

We performed further species‐level analysis of *Pseudomonas*. In APS‐treated mice, only the abundances of *Pseudomonas aeruginosa* , *Pseudomonas stutzeri* , and *Pseudomonas moraviensis* increased (Figure 8G–I), with no statistical significance. This indicates that APS regulates Pseudomonas broadly instead of targeting individual species; the significant genus‐level difference stems from the cumulative effect of coordinated changes in the three species, while the abundance fluctuation of a single species cannot reflect intergroup differences. Notably, despite being an opportunistic pathogen, *Pseudomonas* has been reported to enhance immune cell infiltration in tumor microenvironments and counteract immune evasion [37, 38, 39].

### 3.7 Sterile Supernatants Mixture of Three *Pseudomonas* Species Induced Melanoma Cell Apoptosis and ICD Marker Expression In Vitro

**FIGURE 9:**
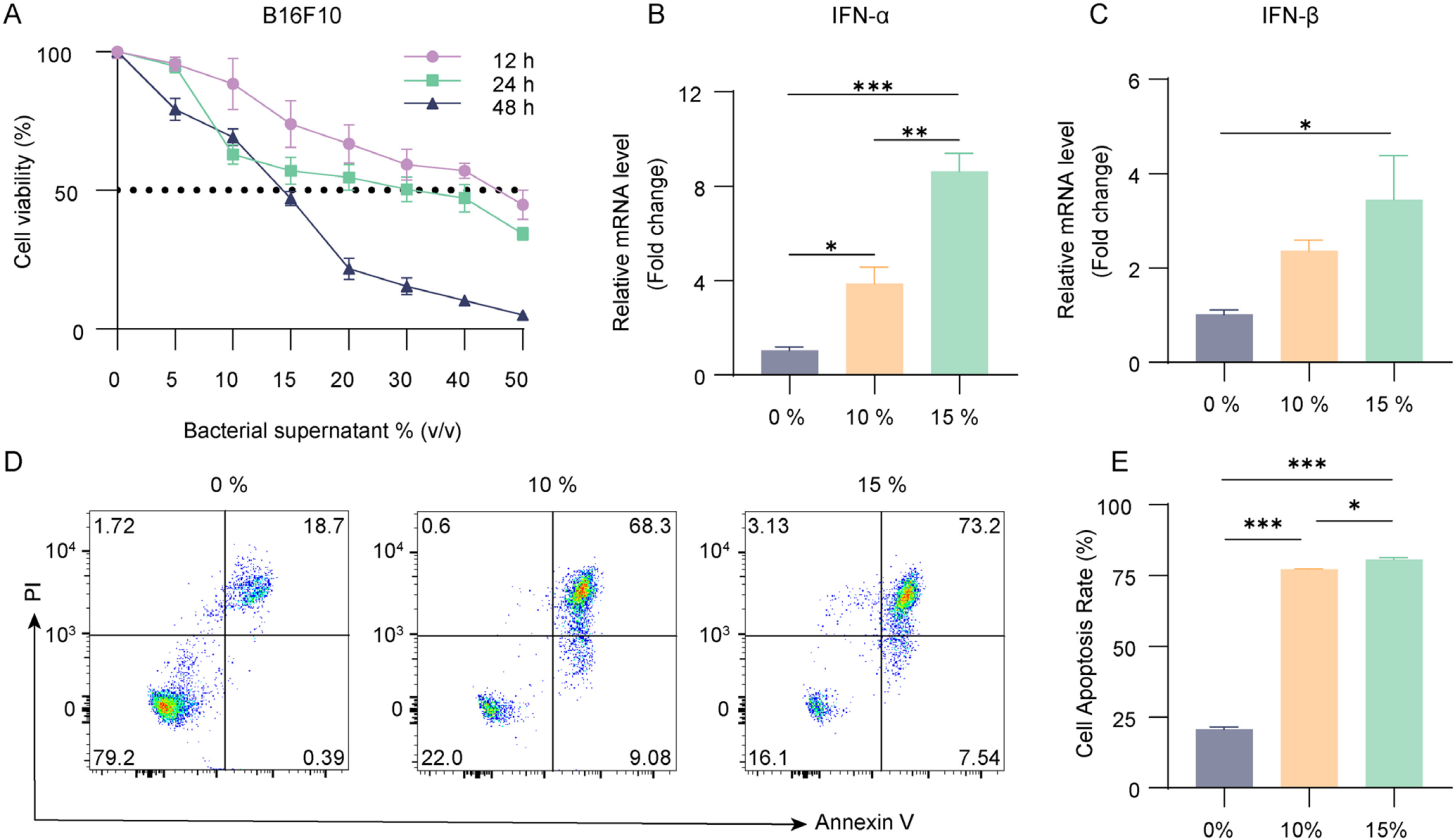
Three *Pseudomonas* species mixed sterile supernatant inhibited melanoma cell growth and induced ICD marker expression. (A) Cell viability of B16F10 cells treated with *Pseudomonas* supernatant mixture was assessed by MTT assay. (B, C) IFN‐α and IFN‐β mRNA expression in treated B16F10 cells measured by RT‐qPCR. (D, E) Flow cytometric analysis of early and late apoptotic proportion following supernatant treatment. Data were presented as mean ± SEM (*n* = 3). *p < 0.05, **p < 0.01, ***p < 0.001.

To further investigate the role of intratumoral *Pseudomonas* in mediating ICD of melanoma cells, we prepared sterile supernatants mixture from three *Pseudomonas* species by filtering crude bacterial supernatants through 0.22 μm nitrocellulose membranes, followed by dilution with culture medium to various concentrations. The mixed supernatant significantly inhibited B16F10 cell proliferation in vitro (Figure 9A). Treatment with the supernatant mixture at concentrations of 10%, 15%, and 20% (v/v) upregulated the mRNA expression of IFN‐α and IFN‐β (Figure 9B,C) and induced apoptosis in B16F10 cells (Figure 9D,E). These findings demonstrated that *Pseudomonas*‐derived sterile supernatant mixture can directly induce melanoma cell apoptosis and stimulate the expression of ICD markers, suggesting its potential role in APS‐induced ICD and antitumor immunity.

### 3.8 *Pseudomonas* Sterile Supernatant Mixture Mediated the cGAS and ICD Activation Triggered by APS in Melanoma

**FIGURE 10:**
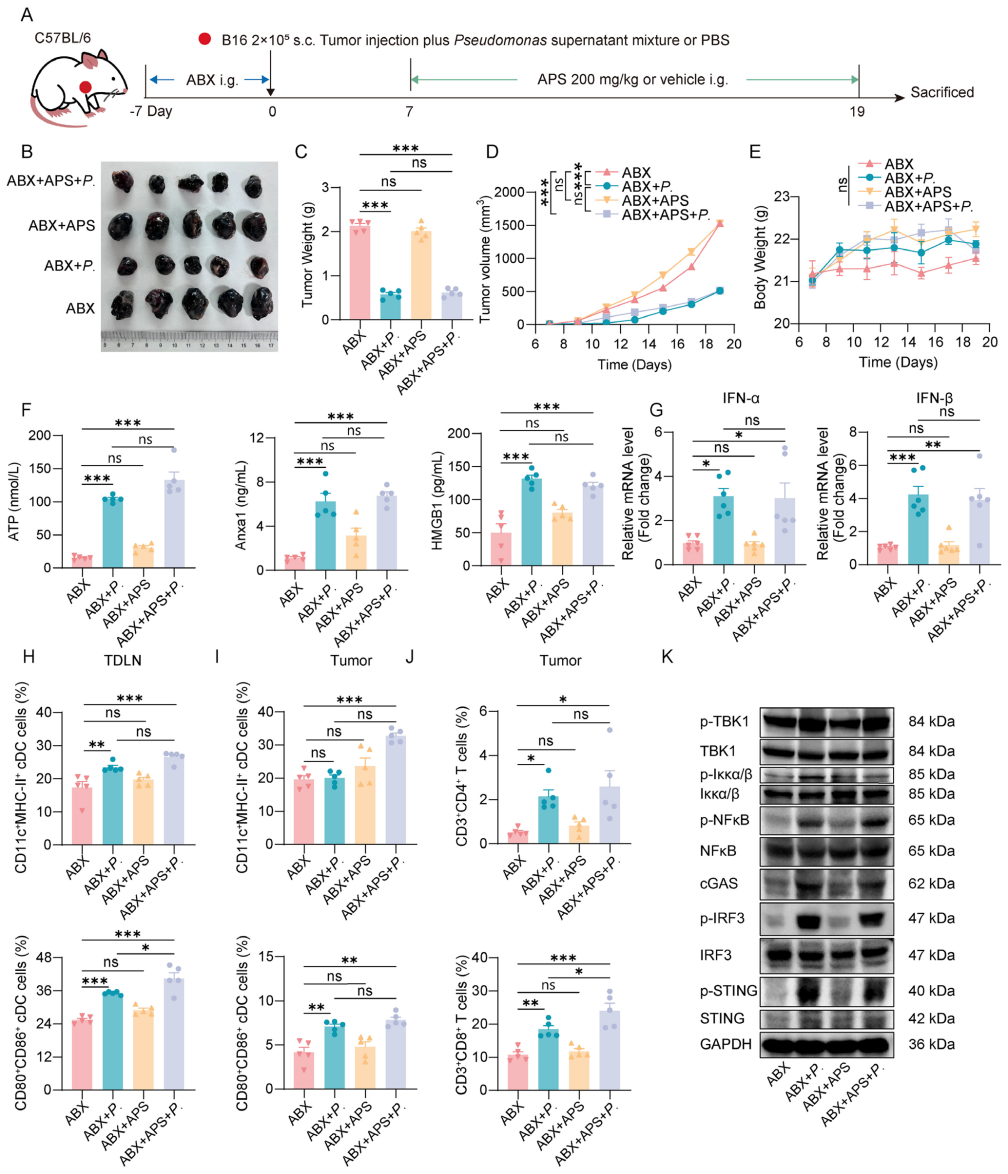
APS‐induced cGAS‐STING and ICD activation in melanoma is dependent on *Pseudomonas* sterile supernatant mixture. (A) In vivo experimental design. (B, C) Representative tumor images and weights collected at 19 days postinoculation. (D) Tumor volume measurements during treatment. (E) Body weight changes across groups. (F) Intratumoral ATP content, serum levels of HMGB1, and Anxa1. (G) IFN‐α and IFN‐β mRNA expression in tumor tissues. (H, I) Flow cytometric quantification of MHC‐II^+^CD11c^+^ DCs and CD80^+^CD86^+^ DCs in TDLNs and tumor tissues. (J) Flow cytometric analysis of CD4^+^ and CD8^+^ T‐cell infiltration in tumor tissues. (K) Western blot detection of cGAS‐STING pathway proteins in melanoma tissues. Data were presented as mean ± SEM (*n* = 5–6). NS, not significant; *p < 0.05, **p < 0.01, ***p < 0.001.

To clarify whether *Pseudomonas* mediates APS‐induced ICD in melanoma, we treated microbiota‐depleted mice with sterile supernatant mixture from three *Pseudomonas* species (Figure 10A). We found that supernatant mixture of the ABX + *P*. group was sufficient to exert antitumor efficacy, reducing tumor volume to level comparable to that of the ABX + APS + *P*. combination therapy group (Figure 10B,C). Both groups showed significantly better outcomes than the ABX group and ABX + APS group (Figure 10D). *Pseudomonas* supernatant mixture also induced cGAS‐STING pathway activation (Figure 10K), enhanced HMGB1 release, upregulated ATP, ANXA1 and IFN‐I expression (Figure 10F,G), promoted DC maturation, and increased CD8^+^ T‐cell infiltration—regardless of concurrent APS administration (Figure 10H,I). These effects were notably higher in the ABX + *P*. and ABX + APS + *P*. groups than in the ABX and ABX + APS groups. Notably, monotherapy with supernatant mixture alone also triggered cGAS‐STING activation and tumor regression, with efficacy comparable to that of APS (200 mg/kg) treatment in wild‐type mice (Figure 1B). This confirms that *Pseudomonas* secretions are key downstream effectors of APS in exerting antitumor response. Collectively, these data indirectly indicate that APS drives antitumor immunity by regulating *Pseudomonas* abundance, while *Pseudomonas* secretions are likely directly responsible for triggering the cGAS‐STING‐ICD cascade.

## 4 Dissussion

Despite progress in melanoma therapy, the prognosis for patients with advanced disease remains poor, underscoring the urgent need for novel therapeutic strategies [40]. ICD represents a promising strategy to enhance tumor immunogenicity and amplify antitumor immunity [41]. APS, a bioactive component with established immunomodulatory properties [42, 43, 44, 45, 46, 47], has shown potential in inhibiting melanoma growth [21, 36]. The antitumor effect of APS is unaffected by its administration [21, 36, 48] or preparation method [49], and ICD induction may be the key mechanism underlying its therapeutic effect. Our findings show that APS induces ICD and triggers antitumor immune responses by remodeling the intratumoral microbiota and activating the cGAS‐STING pathway, identifying the intratumoral microbiota as a key factor in its action.

ICD is a critical determinant of immunotherapy efficacy, driving antitumor immunity through the release of DAMPs (e.g., ATP, CRT, ANXA1, and HMGB1) that facilitate DC maturation and T‐cell priming [3, 4, 50, 51, 52]. Accumulating evidence suggests that certain plant‐derived polysaccharides can effectively trigger this process [53, 54, 55, 56]. Consistent with these precedents and our prior observations [21, 36], this study confirms that APS exerts potent antitumor activity in melanoma by orchestrating a comprehensive ICD profile. Specifically, APS treatment induced significant CRT translocation, extracellular ATP/HMGB1 release, and elevated circulating ANXA1 levels, accompanied by IFN‐I expression. This process enhances CD80/CD86 co‐stimulatory molecule expression on DCs, facilitating T‐cell priming and establishing an immunostimulatory feedback loop [57]. These findings suggest that APS may inhibit melanoma progression through ICD‐mediated immune microenvironment remodeling.

The cGAS‐STING pathway serves as a fundamental cytoplasmic dsDNA sensing mechanism that participates in tumor immunomodulation. A critical finding of our study is the identification of the cGAS‐STING pathway as the downstream sensor mediating APS‐induced ICD. The cytosolic DNA sensing pathway is known to trigger IFN‐I production, bridging innate and adaptive immunity [58, 59]. While this pathway is often silenced in malignancies [60], pharmacological reactivation has been shown to enhance DAMP release and T‐cell recruitment [6, 61]. Our results align with these findings, as APS‐induced tumor suppression and ICD marker expression were diminished by cGAS inhibition with RU.521, indicating that APS acts partly through cGAS–STING activation to elicit ICD.

However, the complexity of APS suggests its effects are likely indirect. Emerging evidence highlights the intratumoral microbiota as a functional regulator of cancer progression and therapy response [62, 63, 64]. Existing evidence indicates intratumoral bacteria can enhance antitumor immunity through bacterial antigen presentation [22], STING activation [25], and immune cell modulation [23, 65]. Using localized antibiotic administration to minimize systemic confounding [66], we provide compelling evidence for a microbial dependency. The observation that antibiotic‐mediated microbiome depletion nullified the therapeutic benefits of APS—including cGAS‐STING activation and ICD induction—indicates that the intratumoral microbiota serves as an indispensable bridge. Sequencing analysis further revealed a specific enrichment of the genus *Pseudomonas* following APS treatment, raising the hypothesis that this taxon may act as a key functional effector.

To further delineate the role of *Pseudomonas*, we conducted rescue experiments using sterile bacterial supernatants. Intriguingly, the administration of *Pseudomonas*‐derived secretory cocktails to antibiotic‐treated mice was sufficient to recapitulate the APS‐mediated phenotype, restoring cGAS‐STING activation and tumor suppression to levels comparable to the original APS treatment. Previous reports have described tumor‐suppressive effects of *Pseudomonas* species via metabolic regulation [39]. In mice, intravenous administration of *P. aeruginosa* in CT26 tumor‐bearing mice induced hemorrhagic necrosis in tumor tissues [67]. *P. aeruginosa* secretes azurin, a copper‐containing redox protein that induces tumor cell apoptosis [37], while *Pseudomonas*‐derived extracellular polysaccharides demonstrate antitumor activity in colorectal cancer models [38]. These findings support a functional role for *Pseudomonas* in APS‐induced ICD and immune modulation.

Given the structural complexity of APS and its dependence on microbial metabolism and host immune interactions, we did not employ in vitro cell models to identify its direct target. While our study substantiates the significant role of *Pseudomonas* in the antitumor effect of APS, we acknowledge that the precise molecular dynamics warrant further dissection. Specifically, although we confirmed that *Pseudomonas* enrichment is necessary for efficacy and that its secretions are sufficient to trigger the pathway, we have not yet distinguished whether APS directly induces secretions to switch to upregulate specific effector molecules, or merely amplifies the signaling intensity by increasing the total bacterial load. Furthermore, given the ecological complexity of the tumor microenvironment, we cannot entirely rule out synergistic contributions from other commensals. Future investigations employing untargeted metabolomics and gnotobiotic (germ‐free) mouse models will be essential to isolate the bioactive compounds and definitively validate causality. Therefore, we did not assert the existence of a direct linear biosynthetic regulatory relationship. Instead, we define the APS‐induced *Pseudomonas* enrichment as a critical functional mediator that creates the necessary microenvironmental conditions for optimal cGAS‐STING activation and antitumor immunity.

**FIGURE 11:**
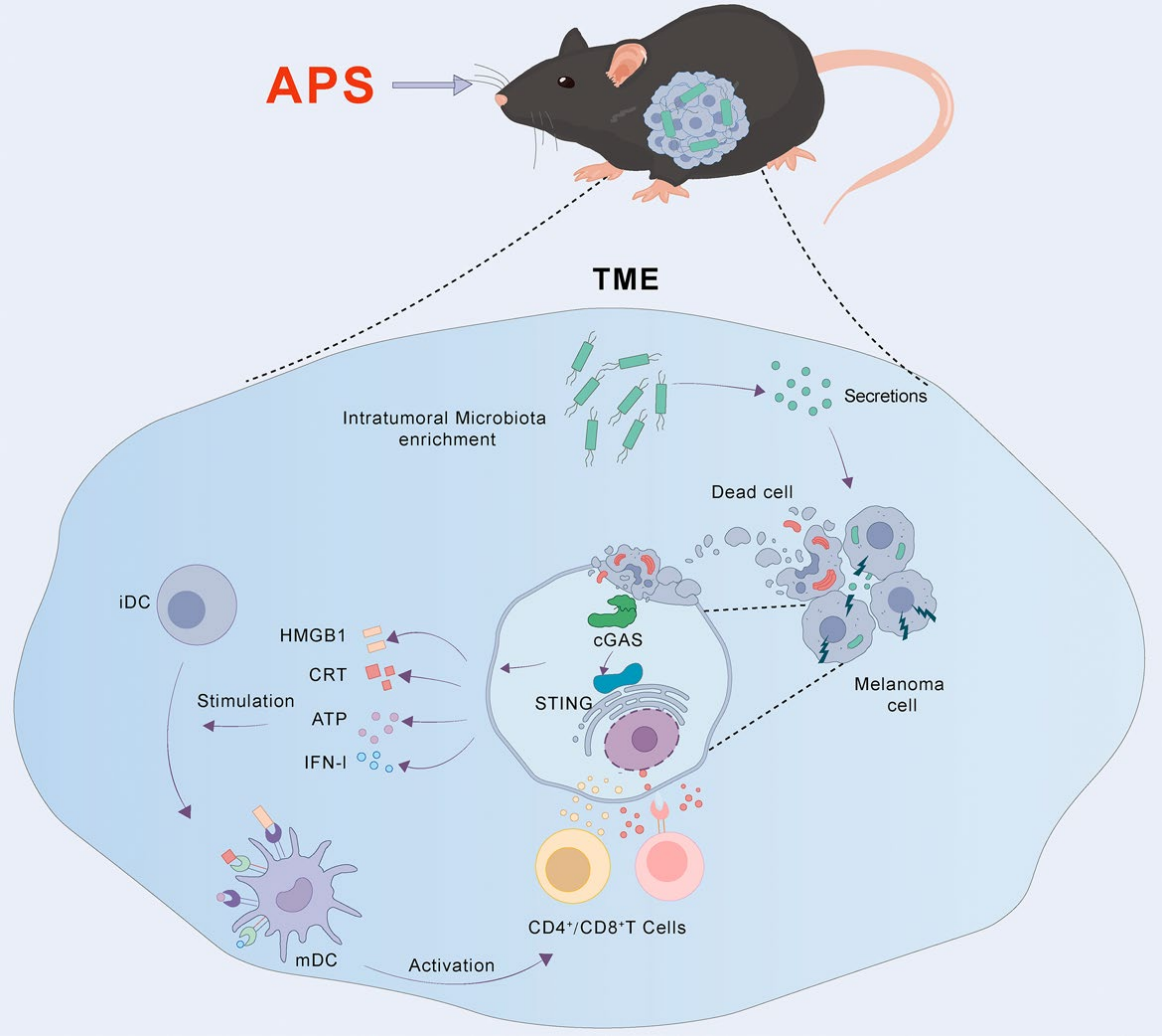
APS exerts its antitumor effects by enriching *Pseudomonas* abundance, activating the cGAS‐STING pathway, inducing ICD in melanoma, promoting DCs maturation, and ultimately activating T lymphocytes.

In summary, APS induces ICD in melanoma by enriching intratumoral *Pseudomonas* and leveraging its secretome to activate the cGAS‐STING pathway. These findings not only elucidate the mechanism of APS but also suggest that targeting the intratumoral microbiota could represent a viable strategy to enhance the efficacy of immunotherapy (Figure 11).

## Author Contributions

**Xiaodong Cheng:** writing – review and editing, supervision. **Guiqing Ding:** investigation, formal analysis. **Yuxin Chen:** resources. **Yuanhua Wang:** resources. **Xiaohan Wang:** resources. **Xi Qiao:** conceptualization, methodology, writing – original draft. **Qijin Lu:** validation, data curation. **Jinyun Ma:** resources.

## Funding

The study was supported by the National Natural Science Foundation of China (No. 82304779) and the Natural Science Foundation of Shanghai (No. 24ZR1467200).

## Ethics Statement

The study was approved by the Laboratory Animal Welfare and Ethics Committee of Yueyang Hospital of Integrative Medicine, Shanghai University of Traditional Chinese Medicine (YYLAC‐2024‐244).

## Consent

The authors have nothing to report.

## Conflicts of Interest

All of the authors of this manuscript are not the current Editor or Editorial Board Member of Cancer Medicine. All authors had full access to all of the data in the study and had final responsibility for the decision to submit for publication. The authors declare no conflicts of interest.

## Supporting information


**Figure S1:** Characterization of APS components by GC–MS analysis. Rha, rhamnose; Fuc, fucose; Ara, arabinose; Xyl, xylose; Man, mannose; Glu, glucose; Gal, galactose.
**Figure S2:** (A) Immunofluorescence staining of LTA and LPS in tumor tissues (scale bar: 200 μm).
**Figure S3:** Analysis of the intratumoral microbiota community levels treated with APS in melanoma mice. *p < 0.05.
**Table S1:** Purity data for APS used in this study.
**Data S1:** Supporting Information.

## Data Availability

The data underlying this study are available from the corresponding author on reasonable request.
