# Supplementary material for: Astragalus Polysaccharides Induce Immunogenic Cell Death in Melanoma: A Mechanism Mediated by cGAS/STING Activation via Intratumoral Microbiota Modulation
Source: Cancer Med. 2026 Apr 14;15(4):e71797. doi: 10.1002/cam4.71797 (PMC13079418; doi:10.1002/cam4.71797)
Supplement: Supplementary file 1 — Figure S1: Characterization of APS components by GC–MS analysis. Rha, rhamnose; Fuc, fucose; Ara, arabinose; Xyl, xylose; Man, mannose; Glu, glucose; Gal, galactose. Figure S2: (A) Immunofluorescence staining of LTA and LPS in tumor tissues (scale bar: 200 μm). Figure S3: Analysis of the intratumoral microbiota community levels treated with APS in melanoma mice. *p < 0.05. Table S1: Purity data for APS used in this study. Data S1: Supporting Information. [file CAM4-15-e71797-s001.zip › cam471797-sup-0001-AppendixS1.docx]

**Supplementary** Table 1. Purity data for APS used in this study.

| Batch | Total sugar content (%) | Residual protein content (%) |
| --- | --- | --- |
| APS- Batch 1 | 65.06 | 10.55 |
| APS- Batch 2 | 60.55 | 8.84 |
| APS- Batch 3 | 63.51 | 11.75 |


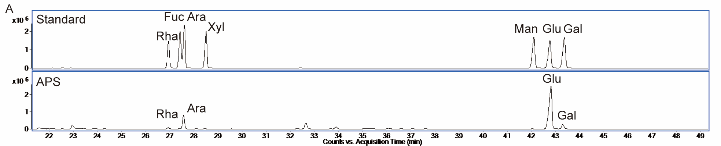


**Supplementary Figure 1**. Characterization of APS components by GC-MS analysis. Rha: rhamnose; Fuc: fucose; Ara: arabinose; Xyl: xylose; Man: mannose; Glu: glucose; Gal: galactose.


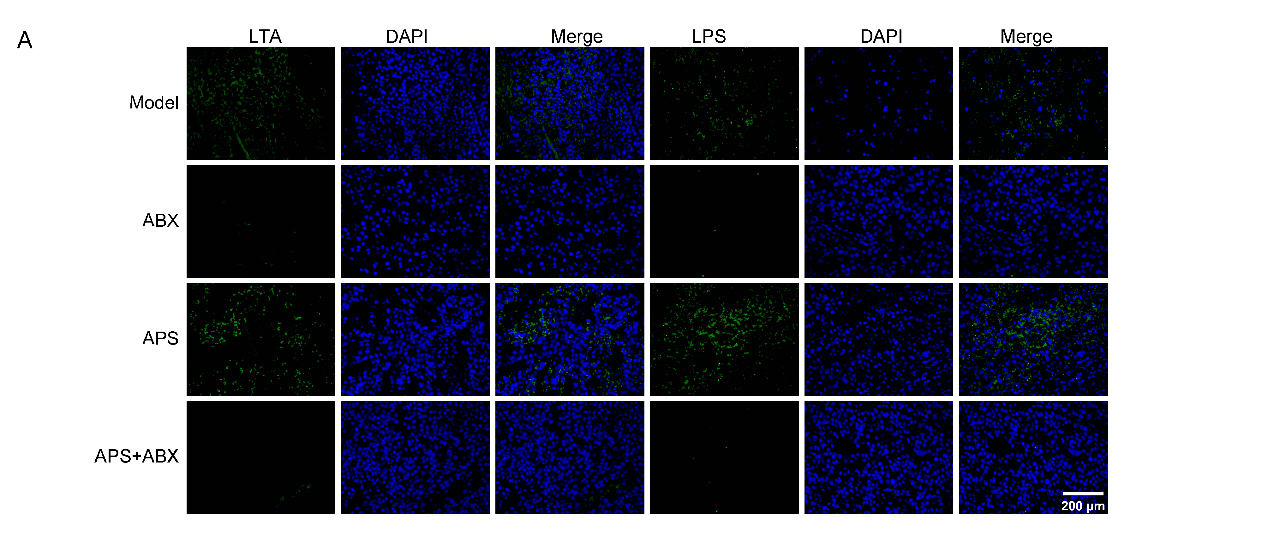


**Supplementary Figure 2**. (A) Immunofluorescence staining of LTA and LPS in tumor tissues (scale bar: 200 μm).


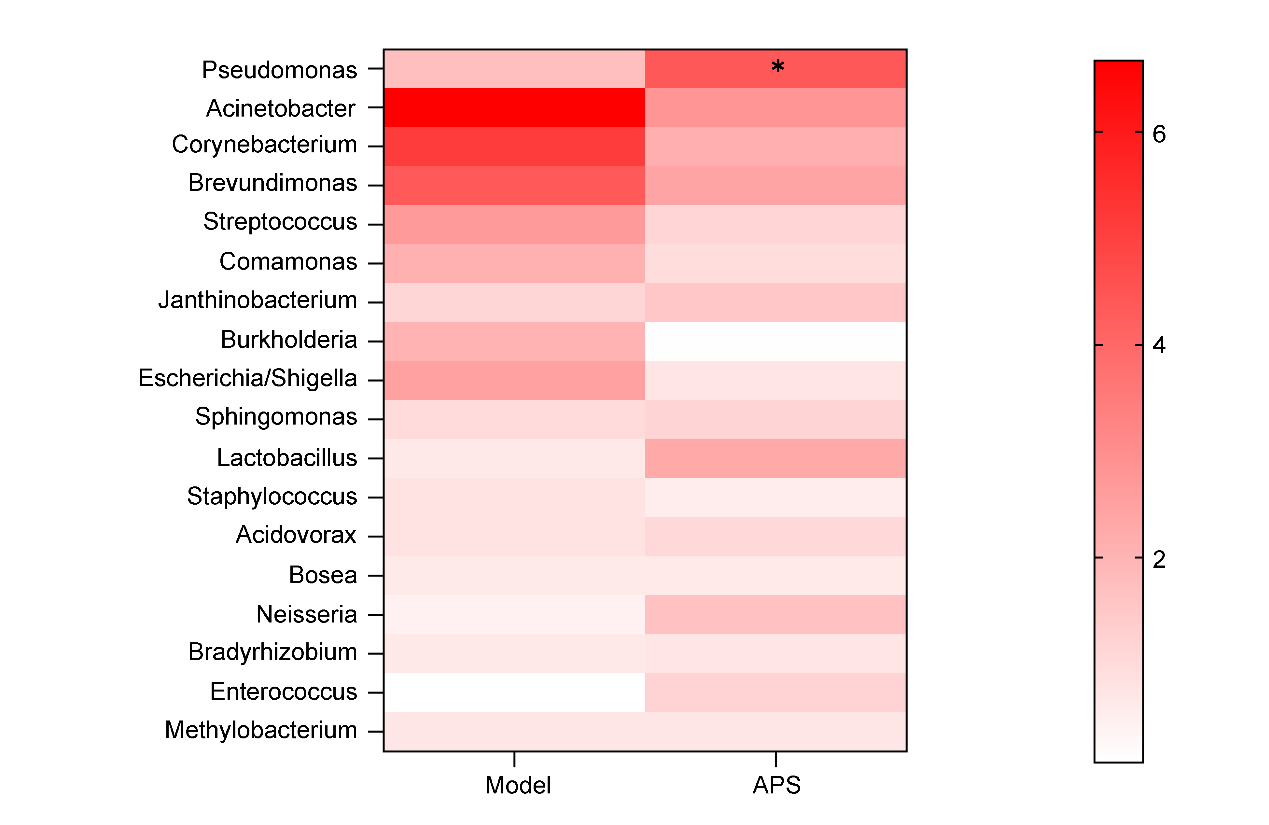


**Supplementary Figure 3**. Analysis of the intratumoral microbiota community levels treated with APS in melanoma mice. ^*^*P*<0.05.
